# Viabahn stent graft compared with prosthetic surgical above-knee bypass in treatment of superficial femoral artery disease: Long-term results of a retrospective analysis

**DOI:** 10.1097/MD.0000000000012449

**Published:** 2018-10-05

**Authors:** Narges Waezi, Shekhar Saha, Ioannis Bougioukas, Alexander Emmert, Bernhard Christoph Danner, Hassina Baraki, Ingo Kutschka, Dieter Zenker, Tomislav Stojanovic, Ahmad Fawad Jebran

**Affiliations:** a Department of Thoracic and Cardiovascular Surgery, University Medical Center, Georg-August University, Göttingen; b Department of Cardiothoracic Surgery, Voelklingen Heart Centre, Völklingen; c Vascular and Endovascular Surgery Hospital Wolfsburg, Wolfsburg, Germany.

**Keywords:** ankle brachial index, endovascular, lower extremities, superficial femoral artery disease, surgical above-knee bypass, Viabahn

## Abstract

The prosthetic surgical above-knee bypass (pAKB) is a standard therapy in superficial femoral artery (SFA) occlusive disease in absence of suitable vein. Viabahn graft has been established as a promising alternative. Since limited comparative data are available, we conducted a retrospective study to compare long-term outcomes of these 2 therapies in a real-world setting.

Records of 52 patients (60 limbs), who were treated by pAKB (29 limbs) or Viabahn (31 limbs) were reviewed. Patients were followed up by clinical assessment, physical examination, and resting ankle brachial index (ABI) after 3, 6, 12 months and yearly thereafter. Long-term data were available for 97% in the Viabahn and 93% for pAKB after 73 ± 3.7 months (mean ± standard error [SE]).

Long-term primary and secondary patencies in Viabahn group were 40% and 70%, respectively, after 63 ± 2.8 months (mean ± SE). Total lesion length was 19 ± 11.06 cm (mean ± SE), graft size was 6 ± 0.72 mm (mean ± SE). Hospital stay was 4.8 ± 0.72 days (mean ± SE). Limb salvage was achieved in 90%. Patients in the pAKB group showed a total lesion length of 24.39 ± 1.97 cm (mean ± SE), graft size was 7 ± 0.99 mm (mean ± SE). Long-term analysis after 83 ± 6.8 months (mean ± SE) revealed a primary patency of 78% with a secondary patency of 94%. Hospital stay was 10.4 ± 1.27 days (mean ± SE). Limb salvage was ensured in 97%. Long-term primary patency was lower for Viabahn (*P* = .044), secondary patency (*P* = .245), and leg salvage (*P* = .389) were not significantly different. However, hospital stay was shorter (*P* = .0002) for Viabahn.

Long-term analysis of Viabahn revealed a significantly lower primary patency, a similar secondary patency, limb salvage, and significantly shorter hospital stay when compared with pAKB. Our data suggest that pAKB is still a valuable option in patients suitable for an open operation. However, Viabahn can be used as a less invasive treatment in high risk patients.

## 1 Introduction

The superficial femoral artery (SFA) and the proximal popliteal artery are the most common anatomic sites of stenosis or occlusion among individuals with peripheral arterial disease.^[1]^ Among traditional treatment strategies the surgical above-knee bypass using autologous vein is still considered the “gold standard” in the treatment of SFA occlusive disease.^[2]^ However, technical advances in endovascular therapy have provided us with alternative treatment tools. After encouraging results of the endovascular implantation of a self-expanding expanded polytetrafluoroethylene (ePTFE)/nitinol stent-graft called Hemobahn endoprosthesis (W. L. Gore, Flagstaff, AZ) from an international trial,^[3]^ the graft was modified and introduced as Viabahn endoprosthesis.^[4]^ Although several subsequent studies since that time confirmed the initial respectable patency results,^[5–10]^ fewer reports exist comparing the outcome of the Viabahn with that of the surgical prosthetic above-knee bypass (pAKB) for the treatment of SFA occlusive disease.^[4,11,12]^ We have previously reported on the early and midterm results of our patient cohort.^[13]^ Studies comparing long-term results after pAKB and endovascular therapy with Viabahn are rather scarce. With our current study we aimed to evaluate and compare the long-term outcome of Viabahn endoprosthesis with the surgical above-knee bypass for the treatment of SFA occlusive disease in a real world setting by retrospective analysis of treated patients by both modalities.

## 2 Materials and methods

### 2.1 Patients

From 2005 to 2011, 52 consecutive patients (60 limbs) who underwent either surgical pAKB or Viabahn endovascular prosthesis implantation in the superficial femoral artery were identified. Written informed consent for study purposes was obtained from all patients prior to treatment start. The medical records of these patients were retrospectively reviewed, including demographic details, risk factors, clinical characteristics, diagnostic images, type of revascularization procedures, and their results. In all patients, conservative therapy was maxed out. All patients suffered from chronic ischemia and were categorized by Rutherford categories based on symptoms and clinical presentation at the time of the revascularization. Patient demographics and associated risk factors are shown in Table 1.

**Table 1:**
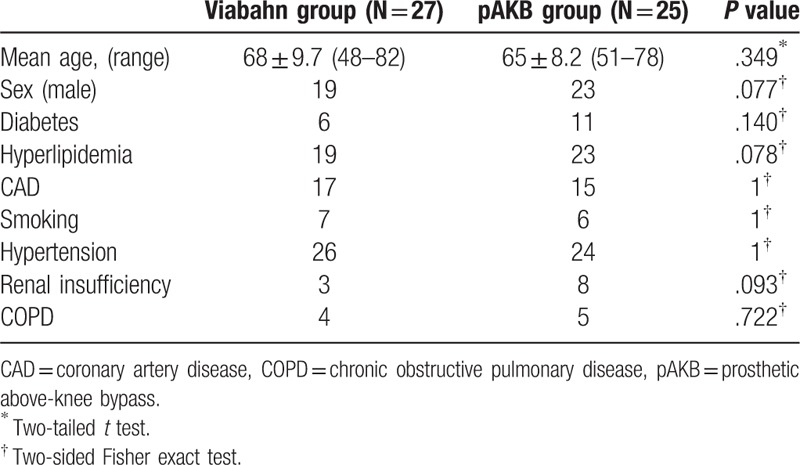
Patient demographics and risk factors.

### 2.2 Technique

Both techniques were performed under general anesthesia. Viabahn stent-graft implantation was performed using standard small surgical exposure of proximal SFA and subsequent traditional angioplasty stenting techniques. The surgical pAKB was accomplished in the usual surgical fashion. As conduit, ePTFE grafts were chosen. All patients received Heparin (5000 Units) during procedure. After treatment combined antiplatelet therapy with aspirin (100 mg/d) and clopidogrel (75 mg/d) was administered for at least 6 months and one of them thereafter in the Viabahn group. Patients in the surgical pAKB group received aspirin 100 mg/d alone. In case of associated comorbidities requiring oral anticoagulation therapy, warfarin was added to the antiplatelet therapy.

### 2.3 Follow-up

Follow-up was performed at 1, 3, 6, 12 months and yearly thereafter and included clinical assessment and resting ankle-brachial index (ABI) measurement. In the case of abnormal findings duplex imaging or CT-Angiography (CTA) was performed to confirm the graft patency. Graft patency was analyzed by primary patency and secondary patency. Primary patency was defined as uninterrupted freedom of restenosis or occlusion within the treated vessel and secondary patency was defined as restored graft patency after occlusion or stenosis.^[14]^ Limb salvage was defined as freedom from a major amputation.

### 2.4 Statistical analysis

In statistical analysis, the two-tailed Fisher exact test was applied to determine differences in patient risk factors and the type of re-intervention. The two-tailed *t* test was used to calculate differences in patient demographics and hospital stay. Generalized Fisher exact test was used to assess differences in grades of limb ischemia, type of lesion according to TASC II (Transatlantic Intersociety Consensus) classification, and number of run-off arteries. The Kaplan–Meier method with the log-rank test was applied to calculate and compare the rates of graft patency, major amputation, and all cause death in each group. A *P* value <.05 was considered statistically significant.

## 3 Results

The retrospective analysis included medical records of 52 patients treated between 2005 and 2011 with either implantation of Viabahn endovascular prosthesis (27 patients, 31 limbs) or femoral to above-knee bypass (25 patients, 29 limbs). No significant difference in demographics and risk factors was found between the 2 treatment groups. Rutherford classification of limb ischemia was used to assess clinical status of patient's limb prior to treatment (Table 2). There were more limbs with higher ischemia grades (grade 4–5) in the Viabahn group. Statistical analysis revealed this difference in Rutherford class distribution to be significant (*P* = .032). Number of lower leg vessels (run-off arteries) was explored on preoperative CT- or magnetic resonance imaging (MRI) scans to assess blood outflow in treated limbs (Table 3). Comparison of run-off arteries in both groups showed a significant difference with a better outflow in the pAKB group. Lesion morphology assessment and typing was performed using TASC II classification recommendations as shown in Table 4. The majority of limbs in the Viabahn group presented with TASC II A and B lesions whereas in the pAKB group most of the lesions represented TASC II C and D. This difference was statistically significant (*P* < .0001). This distribution of lesion types conforms to the recommendations of the TASC II concerning treatment of femoral popliteal lesions.^[15]^ Technical success was achieved in all treated patients. In all, 50 Viabahn endoprosthesis were implanted in 31 limbs, with a mean of 2 ± 0.83 SE grafts per limb. The mean treated lesion length was 19 ± 11.06 cm SE. The mean Viabahn diameter was 6 mm (range, 5–8). In the surgical pAKB group, ePTFE grafts were used in all patients. Patients showed a mean total lesion length of 24.39 ± 1.97 cm SE. Procedural related or early postoperative complications were not noted in both groups. Analysis of hospital stay was performed for both groups and revealed a significantly shorter duration of hospital stay (*P* = .0002) in the Viabahn group with a mean of 4.8 ± 0.75 SE days whereas the mean hospital stay in the surgical group was found to be 9.8 ± 1.05 SE days (Table 5).

**Table 2:**
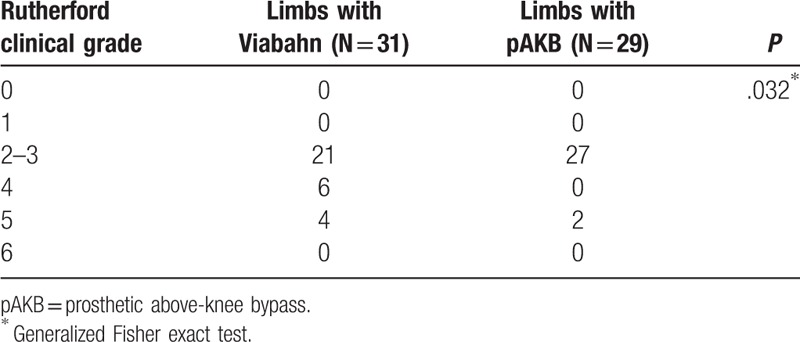
Rutherford clinical grades of chronic limb ischemia in study patients.

**Table 3:**
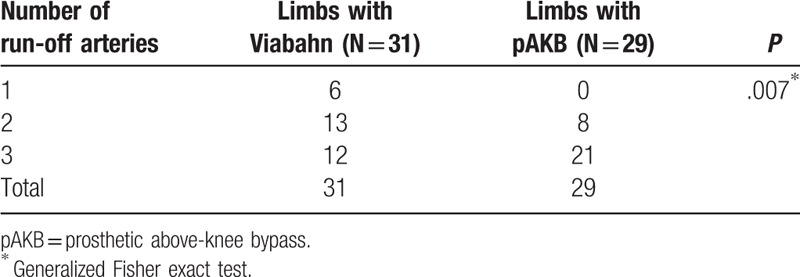
Number of run-off arteries by treatment groups.

**Table 4:**
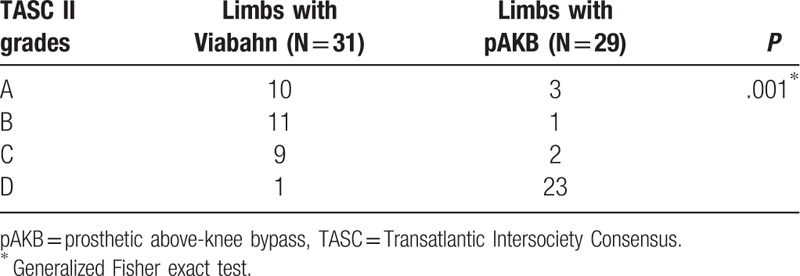
Type of lesion in study patients according to TASC II classification.

**Table 5:**
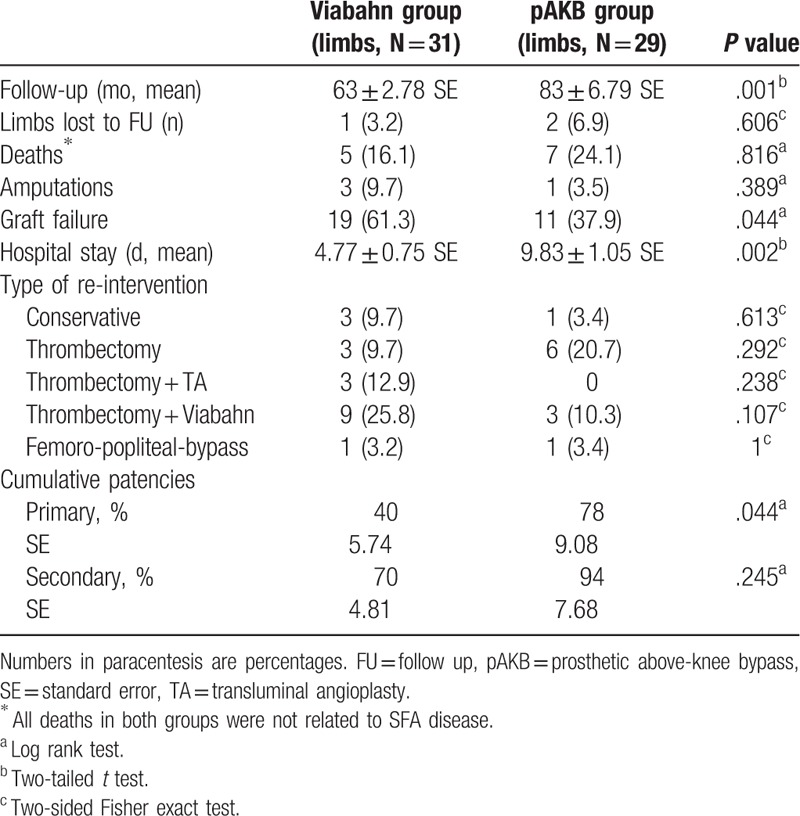
Follow-up data according to treatment groups.

### 3.1 Long-term follow-up

Long-term follow-up data were available for 30 limbs (97%) in the Viabahn group and 27 limbs (93%) in the pAKB group after 73 ± 3.7 SE months (mean ± SE). In the Viabahn group the mean long-term follow-up time was 63 ± 2.78 SE months (Table 5). One patient (1 limb) was lost to follow-up. Five patients died during follow-up. All deaths were not related to SFA disease. Stent-graft failure was observed in 19 limbs (61%) due to thrombosis. There was no Viabahn graft thrombosis during post-procedural hospital stay. Two stent-graft failures occurred during the first month after implantation, 8 stent-grafts failed by the end of the first year after implantation. The remaining 9 stent-graft occlusions happened after a mean period of 34 ± 6.2 months. Among the 19 limbs with failed Viabahn grafts 2 limbs were in Rutherford clinical grade 5 and 4 limbs in Rutherford clinical grade 4, the remaining 13 limbs in Rutherford clinical grade 2 to 3 previous to therapy. The majority of the limbs with graft failure had 3 (9 limbs) or 2 (8 limbs) run-off arteries. One run-off artery was evident in only 2 limbs with occluded grafts. The occluded limbs presented with lesions of TASC II type A (6 limbs), B (7 limbs), C (6 limbs) but not type D prior to treatment. Two patients with occluded grafts had interrupted the antiplatelet therapy because of other surgical procedures. Failed Viabahn grafts were treated with thrombectomy alone in 3 limbs. Additional transluminal angioplasty (3 limbs) or additional implantation of new Viabahn grafts (9 limbs) was required due to progression of the disease or restenosis at the distal edge of the stent-graft. In 1 patient (1 limb) surgical femoro-popliteal bypass to the distal segment of the popliteal artery had to be done. Three limbs were managed conservatively.

In the pAKB group the mean long-term follow-up time was 83 ± 6.79 months (Table 5). One patient (2 limbs) was lost to follow-up. Seven patients expired during follow-up due to reasons not related to SFA disease. In the pAKB group bypass failure due to thrombosis were noted in 11 limbs (38%). None of the graft thrombosis occurred during post-procedural hospital stay. Five bypass grafts failed during the first year after the operation, 1 within the first month, the remaining 4 by the end of the first year after bypass implantation. The remaining 6 bypass graft occlusions took place after a mean period of 47 ± 7.6 months. Among the patients with failed bypasses only 1 patient had suffered from Rutherford clinical grad 5, the remaining patients had shown Rutherford clinical grade 2 to 3 previous to therapy. With respect to the number of run-off arteries, 4 of the affected limbs presented with 2 run-off arteries, 7 limbs in contrast with 3 run-off arteries. Limbs with 1 run-off artery were not affected by graft occlusion. Concerning the TASC II type of lesions, occluded limbs presented with type A (2 limbs), B (1 limb), D (8 limbs) but not type C. Ten of the occluded grafts underwent successfully redo interventions. One patient (1 limb) was treated conservatively. Redo procedures included solely thrombectomy (6 limbs), additional implantation of Viabahn at the level of the distal anastomosis (3 limbs), and surgical femoro-popliteal bypass to the distal segment of the popliteal artery (1 limb).

Major amputation was needed in 3 patients in the Viabahn group (10%) and in 1 patient in the pAKB group (4%) after recurrent graft failure. The difference was not statistically significant (Table 5).

**Figure 1:**
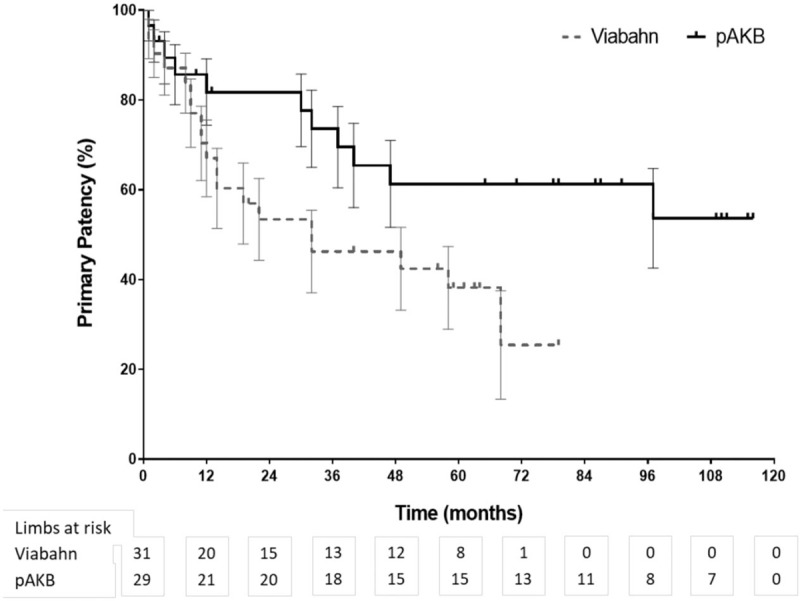
Long-term primary patency during follow-up by Kaplan–Meier analysis (log rank test: *P* = .044).

**Figure 2:**
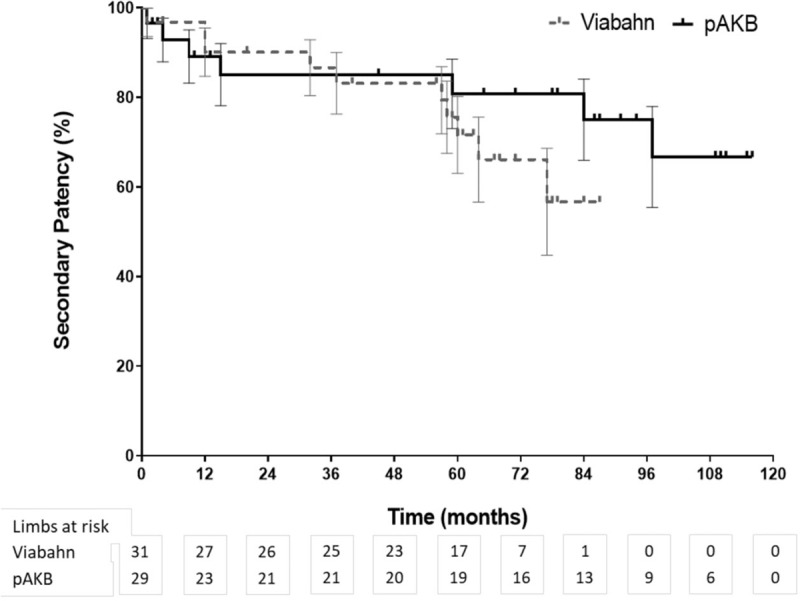
Long-term secondary patency during follow-up by Kaplan–Meier analysis (log rank test: *P* = .245).

**Figure 3:**
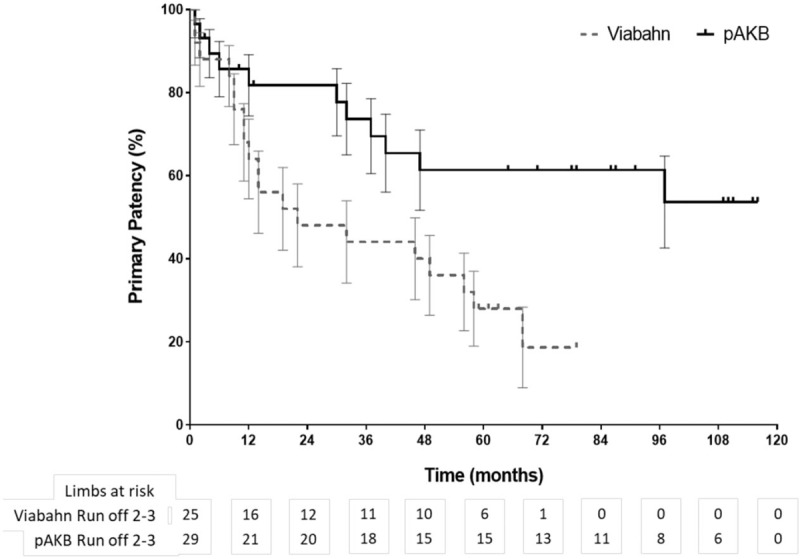
Long-term primary patency during follow-up by Kaplan–Meier analysis according to preoperative number of run-off arteries. Patients with 2 or 3 run-off arteries in each treatment group are compared (log rank test: *P* = .009).

**Figure 4:**
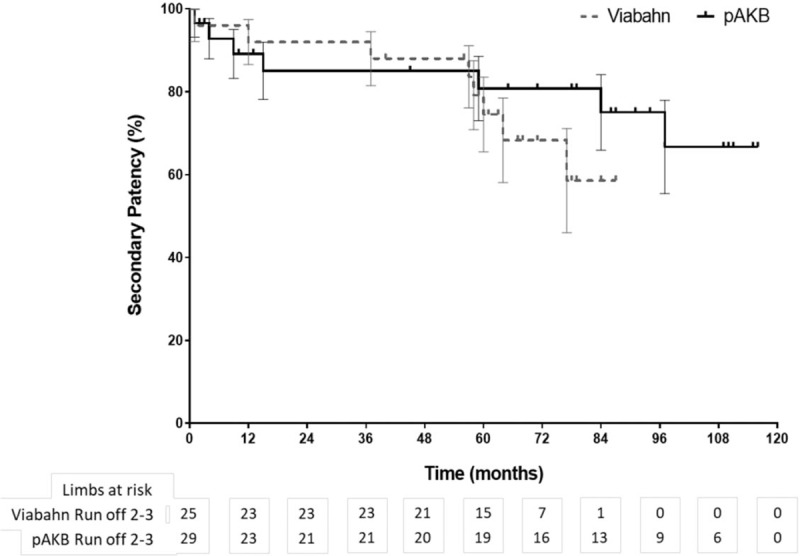
Long-term secondary patency during follow-up by Kaplan–Meier analysis according to preoperative number of run-off arteries. Patients with 2 or 3 run-off arteries in each treatment group are compared (log rank test: *P* = .319).

**Figure 5:**
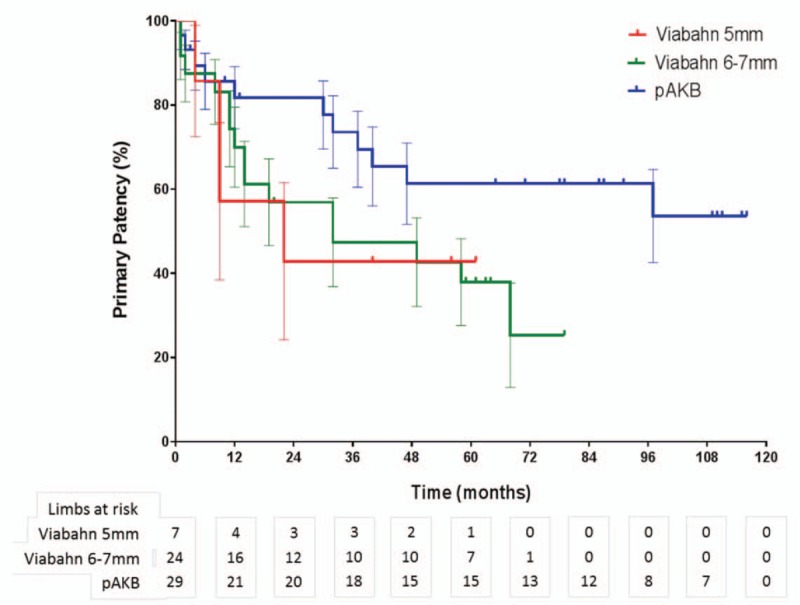
Long-term primary patency during follow-up by Viabahn stent-graft size versus pAK bypass using Kaplan–Meier analysis (log rank test: *P* = .132).

**Figure 6:**
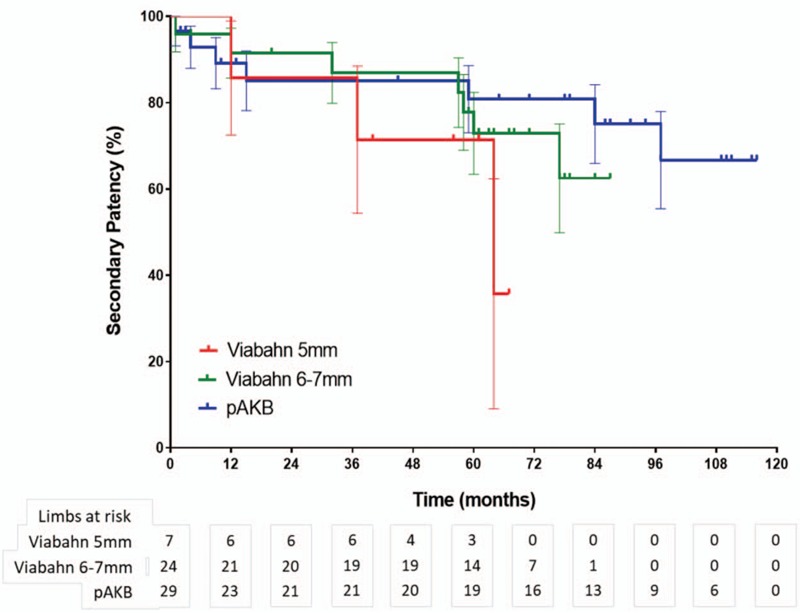
Long-term secondary patency during follow-up by Viabahn stent-graft size versus pAK bypass using Kaplan–Meier analysis (log rank test: *P* = .311).

Primary patency rates calculated by the Kaplan–Meier method were 40% in the Viabahn group and 78% in the pAKB group (Table 5, Fig. 1). This difference proved to be statistically significant (*P* = .044). Secondary patency rates in the Viabahn group did not differ significantly from the pAKB group as shown in Fig. 2 (70% vs 94%, *P* = .245; Table 5). Subgroup analysis was performed to evaluate the influence of various factors such as number of run-off vessels, grade of limb ischemia, TASC II lesion type, and stent size on primary and secondary patency rates in each treatment group and comparing both treatment groups (Tables 6 and 7; Figs. 3–6). This analysis revealed that patients with 2 or 3 run-off vessels who received a pAKB showed a significantly higher primary patency compared with the same cohort of patients in the Viabahn group (78% vs 36%; *P* = .009; Fig. 3). The secondary patency rates were similar in both groups and cohort of patients. No significant differences concerning patency rates were detected dividing the patient cohort into groups according the grade of limb ischemia, TASC II lesion type, and stent size. However, this subgroup analysis has to be looked at cautiously due to an overall small patient cohort and small number of patients in some subgroups.

**Table 6:**
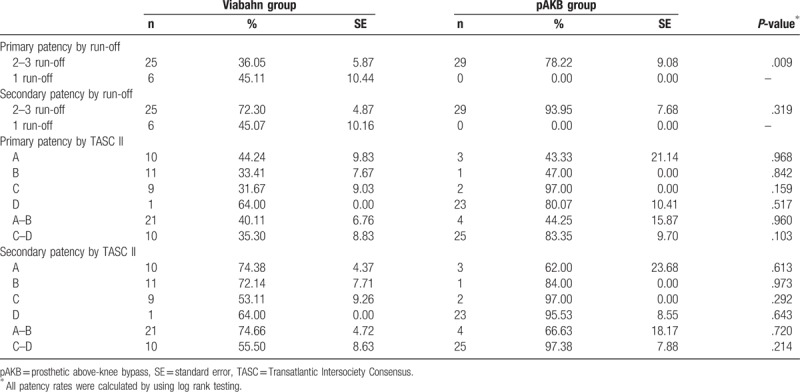
Subgroup analysis of primary and secondary patency rates compared between treatment groups.

**Table 7:**
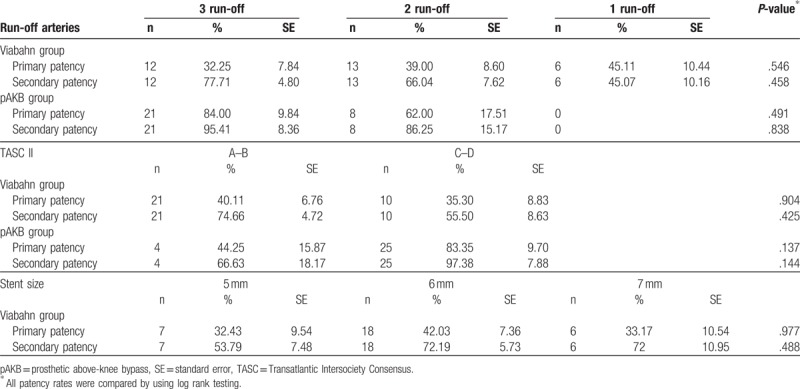
Subgroup analysis of primary and secondary patency rates in each treatment group.

## 4 Discussion

Bypass surgery especially with the use of autologous vein as graft material is still considered the gold standard for SFA disease and recommended therefore as firstline treatment if revascularization is required.^[1]^ Several studies have convincingly demonstrated the superiority of autologous saphenous vein material over prosthetic bypass grafts.^[16–19]^ The later are nevertheless used more often in the daily routine due to several legitimate reasons. The lack of suitable venous material and the idea to preserve the vein for below-knee bypasses, where it evidentially provides superior results compared with synthetic grafts,^[20,21]^ have led to the widespread use of prosthetic bypass material such as Dacron and ePTFE. Among synthetic grafts the use of Dacron or ePTFE has not shown any significant difference in outcome.^[22–24]^ In recent years an evolution in the field of endovascular intervention has taken place. Starting with percutaneous transluminal angioplasty to treat SFA disease with moderate patency results^[25]^ subsequent step-up with self-expanding nitinol stents could provide slightly better results.^[26–28]^ However, late clinical failure caused by in-stent stenosis and stent fracture raised considerable concern.^[29]^ With the introduction of endovascular prosthesis for peripheral occlusive disease (Hemobahn, later Viabahn, W. L. Gore, Flagstaff, AZ) higher patency rates and reduced delayed stent graft failure could be achieved.^[3–7]^ Since reports about comparison of Viabahn graft with pAKB were scarce, we previously published a retrospective analysis of our current patient cohort demonstrating significantly lower primary patency rates for patients treated with the Viabahn stent graft after a mean of 24.5 months.^[13]^ Studies comparing the efficacy of the Viabahn graft with the surgical prosthetic AK bypass for the treatment of SFA occlusive disease on the long run are still very limited. We therefore evaluated long-term data of our patient cohort.

Long-term follow-up data were available for 97% in the Viabahn group and 93% in pAKB group after a mean of 73 ± 3.7 SE months. McQuade et al,^[12]^ who conducted the only so far published long-term study on comparing Viabahn with pAKB for the treatment of SFA occlusive disease, collected follow-up data of 64% and 52% of the patients in the Viabahn and pAKB group after 48 months, respectively. Primary patency rates in the Viabahn group of our patients cohort with a mean lesion length of 15.03 cm were significantly lower compared with the pAKB group (40% vs 78%, *P* = .044), whereas secondary patency rates did not differ significantly (70% vs 94%, *P* = .245). McQuade et al^[12]^ reported in contrast to our results a slightly higher primary patency rate of 48% for Viabahn in patients with a mean lesion length of 25.6 cm and a markedly lower primary patency rate of 58% for pAKB after 48 months. Fischer et al^[6]^ demonstrated long-term primary patency rates of 45% 5 years after implantation of Viabahn grafts in SFA lesions with a mean length of 10.7 cm. Geraghty et al^[30]^ describe in the results of the VIBRANT trial remarkable low primary patency rates of 24.2% 3 years after Viabahn graft implantation in SFA lesions with a mean length of 19 cm. The primary patency rate in the Viabahn group of our study population after a mean of 73 ± 3.7 SE months is comparable with above mentioned studies. In contrast to the limited data on comparing Viabahn with pAKB we conclude a significantly lower primary patency rate for the endovascular graft approach. This could be explained plausibly by a significantly higher Rutherford clinical grade of limb ischemia in our study patients who received a Viabahn graft. Lower leg vessel supply is distributed significantly unequal in our patient cohort. All patients with pAKB had 2 or 3 run-off vessels, whereas the majority of patients in the Viabahn group presented with 1 or 2 run-off vessel supply. But comparing only patients with 2 or 3 run-off arteries in each treatment arm revealed still a significantly lower long-term primary patency for Viabahn graft. The rate of limb loss on the long run was not statistically different in both treatment groups in our study. Size of implanted Viabahn grafts had no impact on the long-term outcome considering patency rates. Both observations are consistent with the analysis of McQuade et al.^[12]^ The hospital stay in the Viabahn group was significantly shorter compared with the pAKB as reported in other investigations.^[11,12]^

Our study has several recognized limitations including the retrospective character, lack of randomization, small total patient number, and a single center experience. However, we could collect long-term data from a high percentage of the treated patients in both groups. This data allow us to report on the efficacy of both the conventional and endovascular approach for the treatment of SFA occlusive disease in real world setting beyond the so far reported time of investigation.

## 5 Conclusion

Our long-term analysis of treatment with Viabahn graft in a real-world setting revealed a significantly lower primary patency rate, when compared with pAKB after 73 ± 3.7 months (mean ± SE) while showing a similar secondary patency rate. Limb salvage is comparable in both treatment groups even after a mean of 73 ± 3.7 months. However, patients treated with Viabahn graft showed a significantly shorter hospital stay. Our data suggest that pAKB is still a valuable option in patients suitable for an open operation. However, the endovascular Viabahn prosthesis can be used as a less invasive approach to treat SFA occlusive disease in high risk surgical candidates and offers a cost-effective therapy due to a reduced hospital stay. Therefore great attention has to be paid to patient selection in the daily clinical routine as graft patency is strongly dependent on patient's cardiovascular risk factors. Our data also suggest that patients with Viabahn grafts should be followed up closely in order to recognize graft failure early and intervene adequately because the secondary patency is acceptable. Further studies are warranted to especially evaluate the role of Viabahn endoprosthesis in patients with complex lesions in comparison to pAKB.

## Acknowledgments

The authors acknowledge support by the German Research Foundation and the Open Access Publication Funds of the Göttingen University.

## Author contributions

**Conceptualization:** Tomislav Stojanovic, Ahmad Fawad Jebran

**Data curation:** Narges Waezi, Shekhar Saha

**Formal analysis:** Narges Waezi, Ahmad Fawad Jebran

**Funding acquisition:** Alexander Emmert, Bernhard Christoph Danner

**Investigation:** Narges Waezi, Shekhar Saha, Ahmad Fawad Jebran.

**Methodology:** Narges Waezi, Shekhar Saha, Ahmad Fawad Jebran

**Project administration:** Dieter Zenker, Alexander Emmert

**Resources:** Hassina Baraki, Ingo Kutschka

**Software:** Ioannis Bougioukas, Alexander Emmert

**Supervision:** Tomislav Stojanovic, Ahmad Fawad Jebran

**Validation:** Tomislav Stojanovic, Ahmad Fawad Jebran

**Visualization:** Narges Waezi, Shekhar Saha, Ahmad Fawad Jebran

**Writing – original draft:** Narges Waezi, Shekhar Saha, Ahmad Fawad Jebran

**Writing – review & editing:** Narges Waezi, Shekhar Saha, Ahmad Fawad Jebran
